# The Role of Anxiety and Depression in Shaping the Sleep–Pain Connection in Patients with Nonspecific Chronic Spinal Pain and Comorbid Insomnia: A Cross-Sectional Analysis

**DOI:** 10.3390/jcm13051452

**Published:** 2024-03-02

**Authors:** Zosia Goossens, Thomas Bilterys, Eveline Van Looveren, Anneleen Malfliet, Mira Meeus, Lieven Danneels, Kelly Ickmans, Barbara Cagnie, Aurore Roland, Maarten Moens, Jo Nijs, Liesbet De Baets, Olivier Mairesse

**Affiliations:** 1 Pain in Motion Research Group (PAIN), Department of Physiotherapy, Human Physiology and Anatomy, Faculty of Physical Education & Physiotherapy, Vrije Universiteit Brussel, Laarbeeklaan 103, 1090 Brussels, Belgium; thomas.bilterys@vub.be (T.B.); eveline.van.looveren@vub.be (E.V.L.); anneleen.malfliet@vub.be (A.M.); mira.meeus@uantwerpen.be (M.M.); kelly.ickmans@vub.be (K.I.); jo.nijs@vub.be (J.N.); 2 Brain, Body and Cognition, Department of Psychology, Faculty of Psychology and Educational Sciences, Vrije Universiteit Brussel, 1050 Brussels, Belgium; aurore.roland@vub.be (A.R.); olivier.mairesse@vub.be (O.M.); 3 Department of Physical Medicine and Physiotherapy, Universitair Ziekenhuis Brussel, Laarbeeklaan 101, 1090 Brussels, Belgium; 4 Institute of Advanced Study, University of Warwick, Coventry CV4 7AL, UK; 5 Department of Psychology, University of Warwick, Coventry CV4 7AL, UK; 6 Department of Rehabilitation Sciences and Physiotherapy, Faculty of Medicine and Health Sciences, Ghent University, Campus Heymans, 9000 Ghent, Belgium; lieven.danneels@ugent.be (L.D.); barbara.cagnie@ugent.be (B.C.); 7 Research Foundation-Flanders (FWO), 1000 Brussels, Belgium; 8 MOVANT Research Group, Department of Rehabilitation Sciences and Physiotherapy, Faculty of Medicine and Health Sciences, University of Antwerp, 2000 Antwerpen, Belgium; 9 Movement & Nutrition for Health & Performance Research Group (MOVE), Department of Movement and Sport Sciences, Faculty of Physical Education and Physiotherapy, Vrije Universiteit Brussel, Pleinlaan 2, 1050 Brussels, Belgium; 10 Brussels University Consultation Center, Department of Psychology, Faculty of Psychology and Educational Sciences, Vrije Universiteit Brussel, 1090 Brussels, Belgium; 11 Department of Neurosurgery, Universitair Ziekenhuis Brussel, 1090 Brussels, Belgium; maarten.ta.moens@vub.be; 12 Department of Radiology, Universitair Ziekenhuis Brussel, 1090 Brussels, Belgium; 13 Center for Neurosciences (C4N), Vrije Universiteit Brussel (VUB), 1090 Brussels, Belgium; 14 Vital Signs and PERformance Monitoring (VIPER), LIFE Department, Royal Military Academy, 1000 Brussels, Belgium; 15 Laboratoire de Psychologie Médicale et Addictologie, CHU/UVC Brugmann, 1020 Brussels, Belgium

**Keywords:** chronic pain, low back pain, network analysis

## Abstract

**(1) Background**: This exploratory study aims to explore the relationship between nonspecific chronic spinal pain (nCSP) and insomnia symptoms, by examining the interconnections, strengths, and directional dependence of the symptoms. In addition, we aim to identify the key symptoms of the nCSP–insomnia relationship and shed light on the bidirectional nature of this relationship. **(2) Methods**: This study is a secondary analysis of the baseline data (cross-sectional) from a randomized controlled trial, which examined the added value of Cognitive Behavioral Therapy for Insomnia (CBT-I) combined with cognition-targeted exercise therapy, conducted in collaboration with the Universiteit Gent and Vrije Universiteit Brussel (Belgium). One hundred and twenty-three nCSP patients with comorbid insomnia were recruited through the participating hospitals, advertisements, announcements in local newspapers, pharmacies, publications from support groups, and primary care. To explore the interconnections and directionality between symptoms and the strengths of the relationships, we estimated a regularized Gaussian graphical model and a directed acyclic graph. **(3) Results**: We found only one direct, but weak, link between sleep and pain, namely, between average pain and difficulties maintaining sleep. **(4) Conclusions**: Despite the lack of strong direct links between sleep and pain, pain and sleep seem to be indirectly linked via anxiety and depression symptoms, acting as presumable mediators in the network of nCSP and comorbid insomnia. Furthermore, feeling slowed down and fatigue emerged as terminal nodes, implying their role as consequences of the network.

## 1. Introduction

Nonspecific chronic spinal pain (nCSP) and insomnia are two common and debilitating conditions that significantly impact an individual’s quality of life [1,2,3]. Furthermore, nCSP is associated with absenteeism leading to high socioeconomic consequences [4]. nCSP, encompassing chronic low back pain, chronic neck pain, and failed back surgery syndrome, is a widespread condition and is defined as chronic if the pain occurs on most days and lasts for at least three months [1,4]. Insomnia, on the other hand, is the most common sleep disorder which manifests as a subjective complaint in initiating, maintaining sleep, and/or waking up sooner than desired. In addition, at least one daytime impairment must be present (e.g., concentration difficulties, mood swings, malaise, sleepiness, or fatigue). Insomnia becomes chronic when it persists for at least three months and occurs at least three nights a week [1,5,6,7]. Insomnia is the most common comorbidity reported among persons with nCSP, and previous research indicates a strong, bidirectional association between nCSP and insomnia [1,7].

Although a reciprocal relationship between pain and sleep seems well accepted [8,9,10,11], multiple studies consider disturbed sleep a better predictor of pain than vice versa [2,8,9,10,11,12]. The contribution of insomnia to the development or amplification of pain is suggested to lie in alterations in pain processing and hyperalgesic responses [8,9]. However, the complexity of the relationship between pain and sleep leaves many remaining questions, such as the potential underlying factors of the direction and strength of the association [12]. These potential factors could lie in prevalent complaints of both persons with insomnia and chronic pain, such as fatigue, symptoms of anxiety, and symptoms of depression [13,14,15]. Although the domain of fatigue and nCSP is less studied, reduction of fatigue is one of the patient-determined success criteria for the treatment of nCSP [16]. On the other hand, symptoms of anxiety and depression are suggested to at least partially mediate the relationship between pain and sleep. Persons with insomnia might be more vulnerable to symptoms of anxiety and depression, as improvement in these symptoms improves sleep disturbance. However, improvements in anxiety and depression do not often predict changes in pain [8,10,12,17,18].

In order to deepen our understanding of the complexity of the relationship between sleep and pain, network approaches should be considered. Such a network approach considers symptoms as constitutive components of a disorder, actively influencing and maintaining each other through direct causal interactions. It uses graph theory to represent symptoms as nodes and their interrelations as edges, which can be visualized and analyzed [19,20]. A Gaussian graphical model (GGM) represents conditional independence associations in an undirected graph. Meanwhile, a cross-sectional directed acyclic graph (DAG) reveals directional dependencies, indicating a stronger influence of one node on another. Integrating these models allows for inferring potential causal relations among nodes [21,22]. Hence, this approach allows us to move beyond the traditional latent variable models and gain a deeper understanding of the dynamic interactions between symptoms of nCSP, insomnia, anxiety, depression, and fatigue [20,23,24,25,26,27].

Given the exploratory opportunities of the network approach, this study aims to provide further insights into the sleep–pain relationship by examining the interconnections, strengths, and directional dependence of symptoms of nCSP, insomnia, anxiety, depression, and fatigue. In addition, we aim to identify the key symptoms in the network. These insights could offer promising avenues for enhancing the overall non-pharmacological management and quality of life for persons suffering both conditions.

## 2. Materials and Methods

### 2.1. Participants

This study is a secondary analysis of the baseline data (cross-sectional) from a randomized controlled trial, which examined the added value of incorporating Cognitive Behavioral Therapy for Insomnia (CBT-I) into the current best physical therapy treatment for nonspecific chronic spinal pain (nCSP). The full study protocol was registered at clinicaltrials.gov (no. NCT03482856) [3]. This study was a multicenter, cross-sectional study, performed by researchers from Vrije Universiteit Brussel and Ghent University. Participants were recruited through the universities and their corresponding hospitals, advertisements, and announcements in local newspapers, pharmacies, publications from patient support groups, and primary care [3]. The selection criteria can be found in Table 1. In total, 123 patients with CSP and comorbid insomnia were included in the study. The definition of insomnia included self-reported sleep difficulties, defined as >30 min of wake during the night [including sleep latency, wake after sleep onset, early morning awakenings, or a combination] for >3 days/week for >6 months, which cause distress or impairment in daytime functioning (despite having adequate opportunity and circumstances to sleep) in the absence of intrinsic sleep disorders and shift work. Polysomnography was used for the identification of intrinsic sleep disorders. Obstructive sleep apnea was defined as an apnea–hypopnea index over 15, and periodic leg movement disorder as a periodic limb movement index over 15. The scoring of these indices followed the American Academy of Sleep Medicine Manual for Scoring of Sleep and Associated Events guidelines [28]. This study was approved by the ethics committee of the Ghent University Hospital (2018/0277) and the University Hospital Brussels (2018/077).

### 2.2. Sociodemographic Information

Age, sex, dominant pain problem (neck pain or low back pain), pain duration, and BMI were collected.

### 2.3. Questionnaires

Participants filled out several questionnaires to assess self-reported sleep such as the Insomnia Severity Index (ISI) [29] and the Pittsburgh Sleep Quality Index (PSQI) [30], as the third edition of the *International Classification of Sleep Disorders* (ICSD-3) and the *International Classification of Diseases*, 11th Revision (ICD-11), recommend a diagnosis of insomnia purely based on subjective complaints [31,32,33]. Fatigue and sleepiness were assessed using the Brugmann Fatigue Scale (BFS) [34] and Epworth Sleepiness Scale (ESS) [35]. Beliefs and attitudes around sleep were assessed using the Dysfunctional Beliefs and Attitudes About Sleep Scale (DBAS) [36]. Pain-related outcomes were captured by the Brief Pain Inventory (BPI) [37] and Central Sensitization Inventory (CSI) [38]. Mental and physical functioning was assessed using the Short Form Health Survey-36 (SF-36) [39]. Additionally, symptoms of anxiety and depression were evaluated using the Hospital Anxiety and Depression Scale (HADS) [40]. The outcomes of items in these questionnaires were used to select nodes for the pain–sleep network.

An item selection of the above-mentioned questionnaires was carried out as the data were collected in the context of a RCT, and a small number of nodes are recommended to increase power, decrease conceptual overlap, and ensure more stable networks. We used a theoretical approach to review all items and selected nodes based on the hypothesis of representing unique constructs [41,42,43]. By employing nodes based on an item level, we avoided topological overlap, which would arise when two nearly similar symptoms would be included and lead to inflated edges in the network [44,45]. An overview of the selected items per questionnaire is provided in Table 2.

To ensure that the selected items were scored in the same direction (e.g., a high score equals a poor outcome) and to reduce bias, we standardized and transformed the selected items [46].

### 2.4. Statistical Analysis

Data were analyzed with R software (version 2023.06.0+421, available at https://r-project.org and version 2022.02.4+500 to run Rgraphviz accessed on 5 September 2023). To estimate, visualize, and measure the stability of the networks, the packages *networktools*, *bootnet*, *ggplot2*, *bnlearn*, *Rgraphviz,* and *qgraph* were used [24,45]. First, we estimated a regularized Gaussian graphical model (GGM) using the graphical least absolute shrinkage and selection operator (gLASSO) combined with the Extended Bayesian Information Criterion (EBIC) [45]. Additionally, the cormethod was set to cor_auto (polychoric correlations), the hyperparameter γ (gamma) was set to 0.5, the number of lambda values tested was 100, the network was not thresholded, and the ratio of lowest lambda value compared to maximal lambda was set to 0.01 [41,45]. The additional analysis included centrality measures (strength and expected influence) with the R package *qgraph*, edge-weights accuracy using bootstrapped confidence intervals (1000 bootstraps), the stability of the edges and centrality measures, the significance of differences between edges within the network using the bootstrapped difference test, bridge symptoms, and bridge centrality and stability measures [24]. Due to some unexpected negative edges, an additional network was estimated using Spearman correlations to compare with the Gaussian graphical model. Two very different networks would indicate an untrustworthy estimation of the polychoric correlations [25]. Second, we estimated a directed acyclic graph (DAG) using a Bayesian hill-climbing algorithm with 50 restarts and 100 perturbations [43,47]. There were no specifications for the edges, meaning that all possible edges were allowed in the network [48].

## 3. Results

### 3.1. Descriptives

The entire sample was included in the network analyses, which comprised 123 persons with nCSP and comorbid insomnia. The mean pain duration of the participants was 90.03 months (*SD* = 96.08). The participants’ age ranged from 21 to 61 years (*M*_age_ = 40.36, *SD*_age_ = 11.06), and the sample included 82 females (67%; *M*_age_ = 39.46, *SD*_age_ = 10.99) and 41 males (33%; *M*_age_ = 42.15, *SD*_age_ = 11.09). The mean BMI was 23.33 (*SD*_BMI_ = 3.14).

### 3.2. Regularized Gaussian Graphical Model

#### 3.2.1. Description

**Figure 1:**
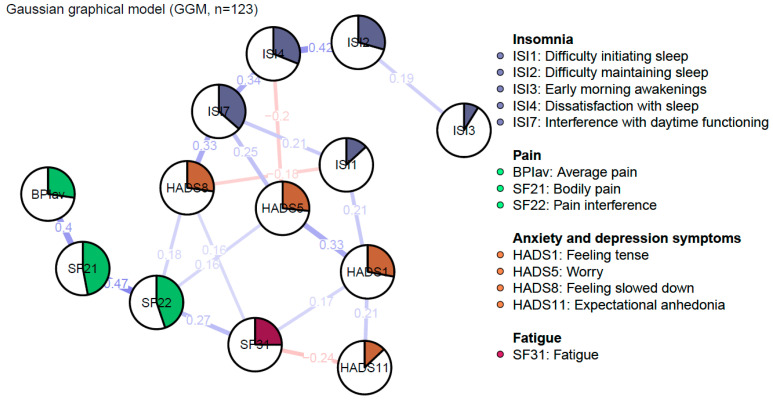
Regularized Gaussian graphical model of pain, insomnia, and affective symptoms: undirected conditional independence associations. The edge’s thickness denotes the magnitude of the pairwise association between nodes (i.e., regularized partial correlations, with a maximum set to 1). Positive associations are visualized with blue edges, while red edges represent negative associations. The pies in the nodes reflect predictability, meaning the proportion of the explained variance of the node by all other nodes in the network.

Figure 1 depicts the regularized GGM of nCSP patients with comorbid insomnia and consists of 13 nodes, divided into four clusters: sleep, pain, fatigue, and anxiety and depression symptoms. The network was sparse due to the gLASSO estimation. The network comprised 28 non-zero edges out of 78 possible edges, which were mainly positive. The mean weight of these edges was rather low (M = 0.05).

#### 3.2.2. Stability

The stability of a network refers to the consistency and resistance to change. Thus, a stable network entails a set of edges, directions, and centrality measures that are unlikely to change considerably [48]. Based on the benchmarks of Epskamp et al. (2018), the stability of the expected influence centrality of the Gaussian graphical model was acceptable, but not ideal (expected influence CS-C = 0.29) [24]. The confidence intervals resulting from the non-parametric bootstrap analysis were rather broad and overlapping. Therefore, the network and the centrality measure should be interpreted with care. Additionally, due to the poor stability of the bridge and strength centrality, they were not interpreted in the present study (bridge strength CS-C = 0.05; bridge expected influence CS-C = 0.05; strength CS-C = 0.05) (see Supplementary Materials).

#### 3.2.3. Predictability

The measure of predictability is the proportion of variance of a node explained by all other nodes in the network, and ranges between zero (the node is not predictable based on other nodes in the network) and one (other nodes of the network can predict the node at hand perfectly). The mean predictability was 0.27. The node “Bodily pain” was the most determined by all other nodes with 46% variance explained (SF21, *R*^2^ = 0.46). Other relatively predictable constructs were “Pain interference” (SF22, *R*^2^ = 0.44), and “Interference with daytime functioning” (ISI7, *R*^2^ = 0.36).

#### 3.2.4. Edges

Edges represent regularized partial correlations between constructs. The strongest edges appeared between nodes of the same cluster. In the pain cluster, the strongest edge emerged between “Bodily pain” and “Pain interference” (SF21–SF22, *r_p_* = 0.47). In the sleep cluster, the strongest edge appeared between “Difficulty maintaining sleep” and “Dissatisfaction with sleep” (ISI2–ISI4, *r_p_* = 0.42). In the anxiety and depression symptoms cluster, the strongest edge appeared between “Tension” and “Worry” (HADS1–HADS5, *r_p_* = 0.33). Other relatively strong edges emerged between “Feeling slowed down” and “Interference with daytime functioning” (HADS8–ISI7, *r_p_* = 0.33), and “Bodily pain” and “Average pain” (SF21–BPIav, *r_p_* = 0.4). However, according to the edge-weights difference test, these edges are not significantly stronger when compared to each other (see Supplementary Materials). Furthermore, there are no direct, or at best weak, edges between the sleep and pain clusters. The clusters seem to be indirectly connected through the anxiety and depression symptoms. Out of these nodes, both sleep and pain variables showed the strongest connection with “Feeling slowed down”. Nevertheless, sleep constructs were more strongly connected in comparison to pain constructs (ISI7–HADS8, *r_p_* = 0.33; SF22–HADS8, *r_p_* = 0.18).

Due to some unexpected negative edges, a Spearman correlation was calculated to evaluate the presence of an artificially induced negative partial correlation via a common effect between “Dissatisfaction with sleep” and “Worry” (ISI4–HADS5), “Feeling slowed down” and “Difficulty initiating sleep” (HADS8–ISI1), and “Fatigue” and “Expectational anhedonia” (SF31–HADS11) [25]. This resulted in a non-significant difference, which means that an artificially induced edge was observed in the regularized partial correlation network between the nodes “Dissatisfaction with sleep” and “Worry” (ISI4–HADS5, *p* = 0.42), “Feeling slowed down” and “Difficulty initiating sleep” (HADS8–ISI1, *p* = 0.61), and “Fatigue” and “Expectational anhedonia” (SF31–HADS11, *p* = 0.31) (see Supplementary Materials).

#### 3.2.5. Centrality Measures

**Figure 2:**
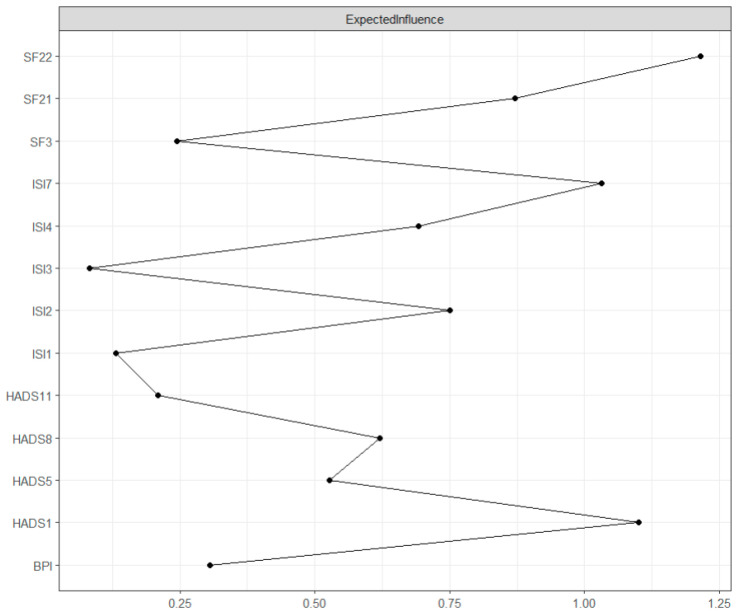
GGM centrality plot: Expected influence. The values on the x-axis represent the unstandardized scores of the centrality measure.

Expected influence is the sum of the value of its connections with other nodes in the network and assesses the node’s influence on its neighboring nodes [21]. As seen in Figure 2, the highest expected influence was found in “Pain interference” (SF22, expected influence = 1.60). Relatively high expected influence was also found in “Tension” (HADS1, expected influence = 1.30), “Interference with daytime functioning” (ISI7, expected influence = 1.12), and “Bodily pain” (SF21, expected influence = 0.70). The centrality difference test indicated that only the nodes with the highest centrality values differed from those with the lowest centrality values (see Supplementary Materials).

### 3.3. Directed Acyclic Graph

**Figure 3:**
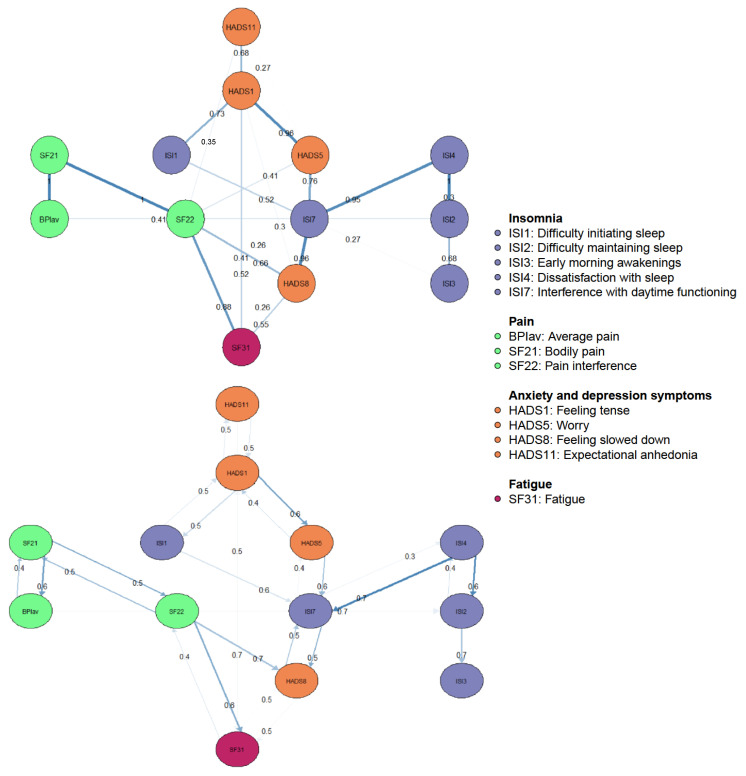
Bayesian networks, directed acyclic graphs: directional dependence relations. In the top panel, edge thickness signifies the magnitude of the Bayesian Information Criterion (BIC). Thicker edges indicate that the removal of the edge would significantly impact model fit. On the panel underneath, edge thickness represents the probability of the direction depicted.

Cross-sectional DAGs reveal directional dependence relations, where the presence of one node more strongly implies the presence of another than vice versa. This enables us to suggest the underlying causal relations between nodes when used in combination with a GGM [21,22]. Figure 3 shows the DAG with the 13 nodes of the pain and sleep network, where only edges greater than 0.25 are depicted. There are several notable features. First, the DAG revealed a chain of constructs dependent on the parent nodes “Expectational anhedonia” and “Tension”. Both showed equal probability of predicting each other, regardless of the direction. While “Tension” appeared to equally predict “Difficulties initiating sleep” and vice versa, it did show a probability of direction to “Worry” greater than 0.5. Therefore, the occurrence of worry is more likely dependent on the presence of tension than vice versa. Second, pain and sleep nodes did not directly predict each other, except for “Average pain” which directly predicted “Difficulty maintaining sleep”. Lastly, “Fatigue” and “Feeling slowed down” are terminal nodes in the DAG, with edges from “Pain interference” and “Interference with daytime functioning”. These terminal nodes showed equal probabilities of direction between each other.

## 4. Discussion

This exploratory study aimed to understand how symptoms of nCSP and insomnia are connected by examining the interconnections, strengths, and directions of symptoms of sleep, pain, fatigue, anxiety and depression. Another aim was to identify the key symptoms, shed light on the bidirectional nature of the nCSP-insomnia relationship, and elucidate whether one condition exacerbates the other.

Previous research showed that sleep quality reduces pain thresholds, and perpetuates pain symptoms and vice versa [2,8,9,10,11,12]. Thus, symptoms can be considered as constitutive components of a disorder, actively influencing and maintaining each other through direct causal interactions [19,20]. We found only one direct link between sleep and pain, namely, between average pain and difficulties maintaining sleep. This amount of connections between sleep and pain symptoms might be explained by important differences between how patients perceive various symptoms and objectively measured symptoms. For example, differences arise in the measurement of sleep when comparing self-reported data and PSG data [49]. Furthermore, the link between average pain and difficulties maintaining sleep was rather weak. The strength of the association is not fully in accordance to previous studies, suggesting a stronger association [8,11]. The strongest links were found between symptoms in the same cluster, which implicates that symptoms have a high probability of covarying because they form an active part of the disorder [20]. Other relatively strong edges emerged between “Feeling slowed down” and “Interference with daytime functioning”. This is in line with the finding that people with insomnia might be more vulnerable to symptoms of anxiety and depression, because contrary to insomnia symptoms, improvement in these symptoms did not predict a change in pain symptoms [8,10,12].

Symptoms of anxiety and depression are suggested to partially mediate the relationship between pain and sleep [14]. In line with this, we found that the pain and sleep clusters were indirectly linked through the cluster of anxiety and depression, suggesting a mediating role. Symptoms of anxiety and depression are more strongly connected to the symptoms of sleep. Also, contrary to the symptoms of sleep, pain symptoms are not situated in the cascade of expectational anhedonia, tension, and worry in the DAGs. Thus, persons with nCSP and comorbid insomnia who score high on the ISI items are more likely to score high on the HADS items. In other words, insomnia symptoms might be the consequence of the variables of the anxiety and depression cluster in the sleep and pain network, while pain symptoms might not be. In addition, all symptoms of anxiety and depression are rather parent nodes instead of the consequence of another node. The only exception is feeling slowed down, which is suggested to be a terminal node in the DAGs with direct connections from both insomnia and pain symptoms (i.e., pain interference and interference with daytime interference due to insomnia). In other words, feeling slowed down might be the consequence of interference in functioning due to both pain and sleep. Here, pain interference might be a greater contributor to feeling slowed down in comparison to interference due to insomnia. This is in line with previous research that suggests that improvements in depression symptoms do not predict change in pain, as the presence of feeling slowed down implies the presence of pain interference more strongly than vice versa [8,10,12]. Besides feeling slowed down, fatigue also appeared as a terminal node, as the presence of fatigue more likely implies the presence of pain interference instead of interference in daytime functioning due to insomnia. This is in agreement with fatigue being a prevalent complaint of both insomnia and chronic pain patients, and with the patient-determined success criterion for the treatment of nCSP being the reduction of fatigue [13,16]. Another possible explanation might be an increase in Type II errors due to the very small sample size in the present study and/or the limited measure of fatigue, in which there is no distinction between mental and physical fatigue [24,41].

Additionally, a network analysis allowed us to reveal the key symptoms in the network. First, the most central constructs in the pain–sleep network were pain interference, bodily pain, interference in daytime functioning, and tension. These constructs were not significantly more central compared to each other in this network. This might be explained by the effect of a small sample size on the accuracy of centrality measures [24]. Nevertheless, when a construct is connected to many nodes but only explains a little of the variance in the network, this construct might not be as important to the network as a construct which is connected to only two other constructs but explains half of the variance in the network [21]. In this network, the most central constructs, except for tension, were most determined by all other constructs in the network, which suggests some importance to the network, which is, however, limited due to the lack of a large sample size. Furthermore, it is important to note that the networks do not include all possible variables. Missing nodes include stressors other than pain, predisposing factors for insomnia (e.g., arousal predisposition), fear of pain, etc. These and other “etiological nodes” are unobserved and thus latent in this network structure [20]. Although they probably influence other variables, they do not form an unobserved variable that influences all other variables. Thus, they do not qualify to portray latent variables as implied in the latent variable model [20].

Previous research showed a reciprocal relation between sleep disturbance and chronic pain, where sleep quality reduces pain thresholds, and perpetuates pain symptoms and vice versa [2,8,9,10,11,12]. Nonetheless, sleep disturbance is considered a better predictor of pain than vice versa. This is suggested to lie in the contribution of insomnia in the abnormalities in pain processing and hyper analgesic responses [8,9]. Contrary to the cited research, we found only one direct, but weak, link between sleep and pain, in which pain is more strongly connected to sleep compared to the other way around. This means that the difficulties maintaining sleep might be a consequence of the presence of average pain in nCSP patients with comorbid insomnia [21]. In other words, if difficulties maintaining sleep are present, it more strongly implies the presence of average pain than vice versa. One potential explanation for the lack of strong, direct associations between sleep and pain might be that anxiety, depression, and fatigue mediate the link between insomnia and nCSP [10,12,13]. Another possible explanation is that questionnaires assess the subjective experience of symptoms, which is different from the objective measurements of these symptoms, and might be subject to reporting biases [50,51].

Moreover, the symptoms of anxiety and depression are parent nodes instead of the consequence of another node. Only feeling slowed down might be the consequence of interference in functioning due to both pain and sleep. This aligns with the vulnerability model of tonic/phasic dopamine dysregulation, suggesting that irregularities in the mesolimbic dopamine system trigger insomnia, chronic pain, and depression symptoms [18]. Exacerbations of these symptoms create a feedback loop, which further contributes to dopamine dysregulation [14].

### 4.1. Strengths and Limitations

This study has several strengths. Firstly, to our knowledge, no research using network analysis on an item level and directed acyclic graphs has been applied to assess comorbidity between nCSP and insomnia. This could potentially inspire further studies to examine the interrelations within a larger sample size, which is a crucial step in confirming the robustness and generalizability of our initial findings. Moreover, we used standardized questionnaires. Nevertheless, some limitations of our study can be mentioned. First, in terms of network analyses, our sample size is limited. This may have led to poor or acceptable (but not ideal) stability [24]. Therefore, the interpretation of this explorative study should be performed with care. Second, cross-sectional DAGs can only disclose directional dependence relations, but cannot confirm temporal precedence [22]. Third, the assumption of causal sufficiency and causal faithfulness in DAGs were violated in this study. In other words, not all common causes are integrated in the network [48]. This means that the estimated causal effects may be biased [48]. Adhering to these assumptions is challenging in psychological data, as it is difficult to retain information in questionnaires on the directionality of influence between symptoms and if there are latent variables underlying these symptoms [48]. Therefore, these assumptions, with the causal faithfulness in particular, are likely to be violated [48].

#### Implications

These directional dependence relations among symptoms should not be mistaken for causal relations either. Consequently, straightforward implications for treatment in individuals with nonspecific chronic spinal pain and comorbid insomnia are difficult to obtain. Early research on network analysis suggested that symptoms high on strength centrality could be potential therapeutic targets for intervention [52,53]. Later work on network analysis urged to reconsider this, as drawing this conclusion comes with caveats [54]. Therefore, our presented results are best understood as a step toward theories about the structure of the causal system underlying nonspecific chronic spinal pain and comorbid insomnia [22]. In other words, these results could be seen as simplified, preliminary depictions of potential causal associations, which, in turn, could lead to clearer, straightforward implications for diagnosis, assessment, and treatment [22].

## 5. Conclusions

The current study explored the interrelationships between pain and sleep in nonspecific chronic spinal pain patients with comorbid insomnia, identifying key symptoms and highlighting the role of anxiety, depression, and fatigue. Contrary to previous research, no direct, or at best weak, links were found between sleep and pain, except average pain and difficulties maintaining sleep. Anxiety and depression were more strongly connected to sleep compared to pain, suggesting their influence in the sleep–pain relationship. Furthermore, feeling slowed down and fatigue emerged as terminal nodes, also implying their role as consequences of the network. Overall, we contributed insights into the interrelationship between sleep and pain. Future studies could further explore the interrelations in nonspecific chronic spinal pain patients.

## Figures and Tables

**Table 1:** Selection criteria.

Inclusion	Exclusion
Aged between 18 and 65 years	Body Mass Index > 30, since this study used the baseline data of an RCT investigating an intervention
Native Dutch speaker	Being diagnosed with chronic widespread pain syndrome (e.g., fibromyalgia and chronic fatigue syndrome)
Experiencing nonspecific spinal pain for at least 3 months, at least 3 days/week, including chronic low back pain (CLBP), failed back surgery syndrome (i.e., surgery more than 3 years ago and anatomically successful surgery without symptom disappearance), and chronic traumatic and nontraumatic neck pain	Thoracic pain in the absence of neck or low back pain Neuropathic pain
Experiencing insomnia: self-reported sleep difficulties defined as >30 min of wake during the night [including sleep latency, wake after sleep onset, early morning awakenings, or a combination] for >3 days/week for >6 months, which cause distress or impairment in daytime functioning (despite having adequate opportunity and circumstances to sleep) in the absence of intrinsic sleep disorders and shift work	History of specific spinal surgery (i.e., surgery for spinal stenosis) to ensure the exclusion of degenerative (joint) diseases.
Not undertaking exercise (>3 metabolic equivalents) 3 days before the assessments	Severe underlying sleep pathology (identified through polysomnography), This includes sleep apnea (AHI > 15) and periodic limb movement disorder (>15/h).
Refraining from analgesics, caffeine, alcohol, or nicotine for 48 h before the assessments, since this study used the baseline data of an RCT investigating an intervention	Shift workers
Willing to participate in therapy sessions and not allowed to continue any other therapies (i.e., other physical therapy treatments, acupuncture, osteopathy, etc.), except for usual medication; and not having received any form of pain neuroscience education or sleep training before	Being pregnant or being a parent within one year post partum
Not starting new treatments or medication and continuing their usual care 6 weeks before and during study participation (to obtain a steady state)	Presence of a current clinical depression diagnosed by a doctor
	Suffering from any specific medical condition possibly related to their pain (e.g., neuropathic pain, a history of neck or back surgery in the past 3 years, osteoporotic vertebral fractures, and rheumatologic diseases)
	People living more than 50 km away from the treatment location were excluded to avoid dropout because of practical considerations.

**Table 2:** Item selection.

Number	Variable Name	Question	Answer Options
1	ISI1	Difficulty falling asleep?	None/mild/moderate/severe/very severe
2	ISI2	Difficulty staying asleep?	None/mild/moderate/severe/very severe
3	ISI3	Problems waking up too early?	None/mild/moderate/severe/very severe
4	ISI4	How satisfied/dissatisfied are you with your current sleep pattern?	Very satisfied/satisfied/neutral/dissatisfied/very dissatisfied
5	ISI7	To what extent do you consider your sleep problem to interfere with your daily functioning (e.g., daytime fatigue, mood, ability to function at work, daily chores, concentration, memory, mood, etc.) currently?	Not at all interfering/a little/somewhat/much/very much interfering
6	BPIav	Please rate your pain by marking the box beside the number that best describes your pain on average.	0 (No pain)–10 (pain as bad as you can imagine)
7	SF21	How much bodily pain have you had during the past 4 weeks?	Not at all/slightly/moderately/severe/very severe
8	SF22	During the past 4 weeks, how much did pain interfere with your normal work (including both work outside the home and housework)?	Not at all/a little bit/moderately/quite a bit/extremely
9	HADS1	In the past week I have been feeling tense or ‘wound up’	0 (Not at all)–3 (most of the time)
10	HADS5	In the past week I had worrying thoughts go through my mind	0 (Only occasionally)–3 (a great deal of the time)
11	HADS8	In the past week I have been feeling as if I am slowed down	0 (Not at all)–3 (nearly all the time)
12	HADS11	In the past week I have been looking forward with enjoyment to things	0 (As much as I ever did)–3 (hardly at all)
13	SF31	Did you feel tired?	All of the time/most of the time/some of the time/a little bit of the time/none of the time

Legend: ISI, Insomnia Severity Index; BPI, Brief Pain Inventory; SF, Short Form Health Survey-36; HADS, Hospital Anxiety and Depression Scale. Numbers represent the items in the survey.

## Data Availability

No new data were created or analyzed in this study. Data sharing is not applicable to this article.

## Supplementary Materials

The following supporting information can be downloaded at: https://www.mdpi.com/article/10.3390/jcm13051452/s1, Table S1: Edge-weights matrix: regularized GGM; Figure S1: Edge-weights stability graph: GGM; Figure S2: Centrality stability graph: GGM; Figure S3: Edge-weights difference test: GGM; Figure S4: Spearman correlation network; Figure S5: Centrality difference test: GGM.
