# Supplementary material for: The Role of Anxiety and Depression in Shaping the Sleep–Pain Connection in Patients with Nonspecific Chronic Spinal Pain and Comorbid Insomnia: A Cross-Sectional Analysis
Source: J Clin Med. 2024 Mar 2;13(5):1452. doi: 10.3390/jcm13051452 (PMC10932262; doi:10.3390/jcm13051452)
Supplement: Supplementary file 1 [file jcm-13-01452-s001.zip › Supplementary Figure S1.pdf]

Figure S1

Edge weights stability graph: GGM

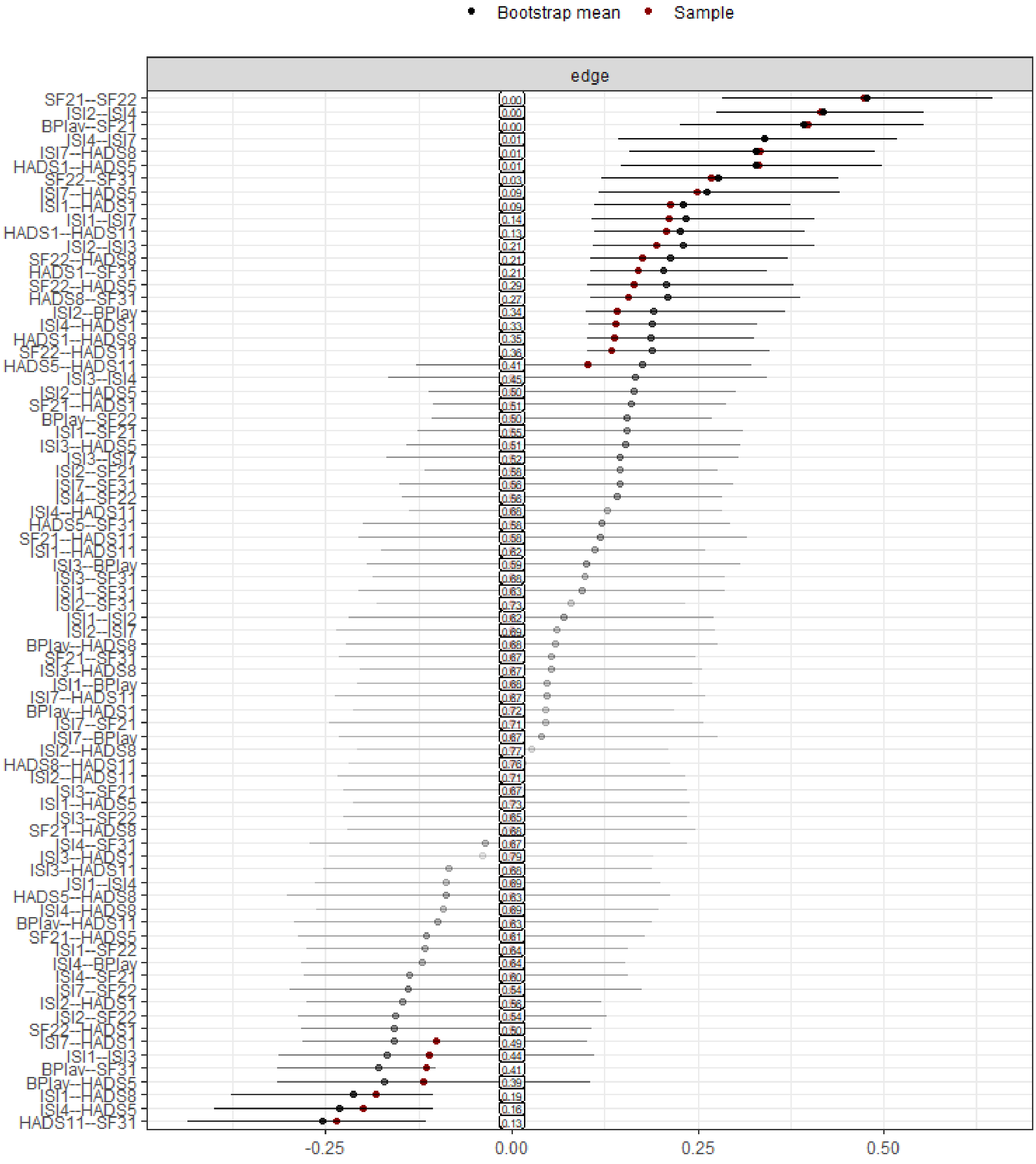

Note. Bootstrapped confidence intervals (CI) of estimated edge-weights. The lines represent the bootstrapped CIs. Every horizontal line represents one edge of the network and is ordered from highest edge weight to lowest. The red dots represent the sample edge weight estimates. The black dots represent the bootstrapped mean values of the edge weights. The numbers indicate the proportion of the edge-weight being excluded in the bootstraps.
