# Supplementary material for: The Role of Anxiety and Depression in Shaping the Sleep–Pain Connection in Patients with Nonspecific Chronic Spinal Pain and Comorbid Insomnia: A Cross-Sectional Analysis
Source: J Clin Med. 2024 Mar 2;13(5):1452. doi: 10.3390/jcm13051452 (PMC10932262; doi:10.3390/jcm13051452)
Supplement: Supplementary file 1 [file jcm-13-01452-s001.zip › Supplementary Figure S3.pdf]

Figure S3

Edge-weights difference test: GGM

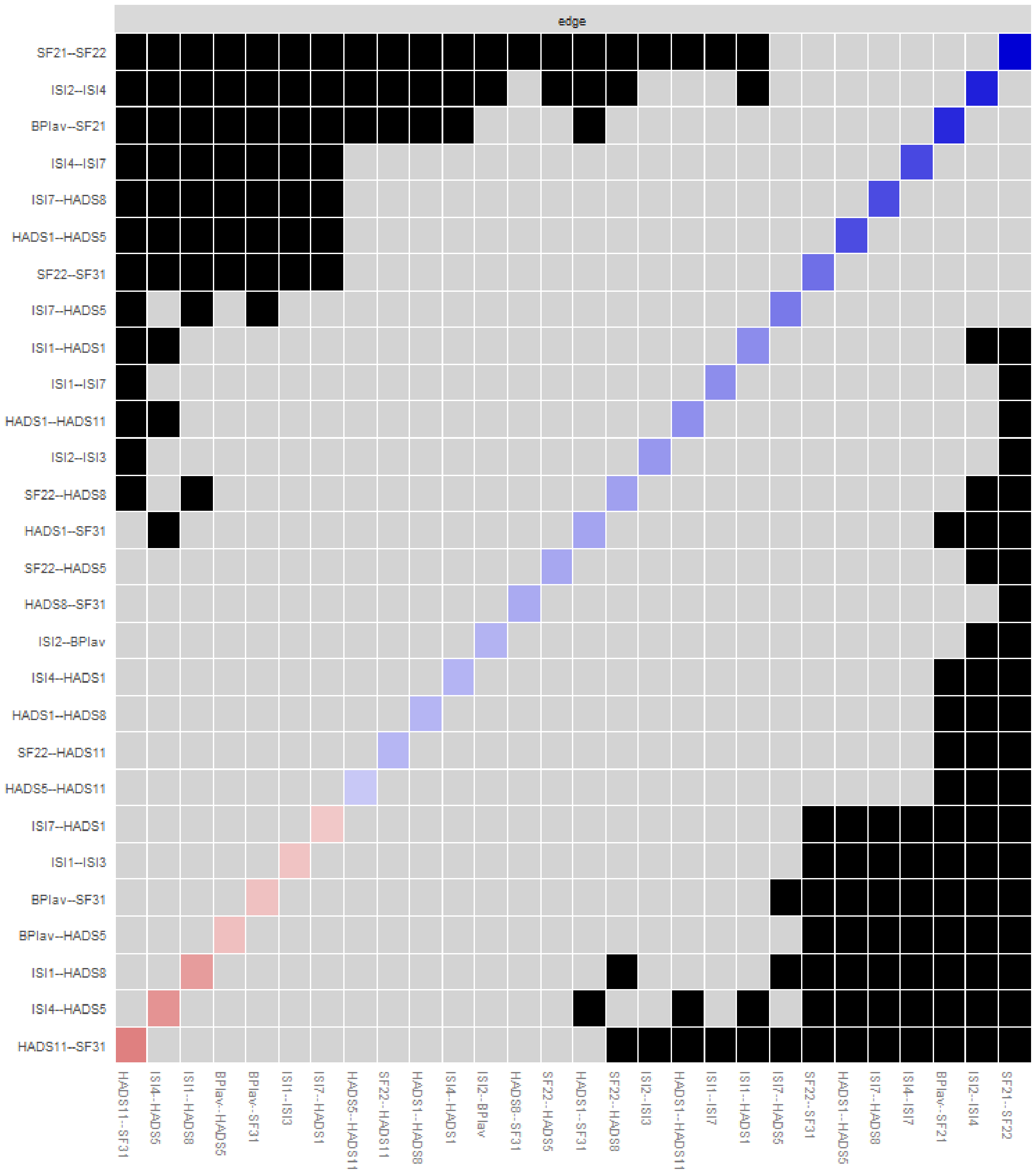

Note. The bootstrapped difference tests (α = 0.05) between edge-weights in the estimated network. Only edge-weights that were non-zero are included. Gray boxes point out edges that do not differ significantly from each other. Black boxes point out edges that do differ significantly from each other. The colored boxes represent the color of the edge as presented in the regularized partial correlation network.
