# Supplementary material for: The Role of Anxiety and Depression in Shaping the Sleep–Pain Connection in Patients with Nonspecific Chronic Spinal Pain and Comorbid Insomnia: A Cross-Sectional Analysis
Source: J Clin Med. 2024 Mar 2;13(5):1452. doi: 10.3390/jcm13051452 (PMC10932262; doi:10.3390/jcm13051452)
Supplement: Supplementary file 1 [file jcm-13-01452-s001.zip › Supplementary Table S1.pdf]

Table S1

Edge weights matrix: regularized GGM

	ISI1	ISI2	ISI3	ISI4	ISI7	BPI	SF21	SF22	HADS1	HADS5	HADS8	HADS11	SF31
ISI1	.00												
ISI2	.00	.00											
ISI3	-.11	.19	.00										
ISI4	.00	.42	.00	.00									
ISI7	.21	.00	.00	.34	.00								
BPI	.00	.14	.00	.00	.00	.00							
SF21	.00	.00	.00	.00	.00	.40	.00						
SF22	.00	.00	.00	.00	.00	.00	.47	.00					
HADS1	.21	.00	.00	.14	-.10	.00	.00	.00	.00				
HADS5	.00	.00	.00	-.20	.25	-.12	.00	.16	.33	.00			
HADS8	-.18	.00	.00	.00	.33	.00	.00	.18	.14	.00	.00		
HADS11	.00	.00	.00	.00	.00	.00	.00	.13	.21	.10	.00	.00	
SF31	.00	.00	.00	.00	.00	-.12	.00	.27	.17	.00	.16	-.24	.00
