# Supplementary figures and images for: The Role of Anxiety and Depression in Shaping the Sleep–Pain Connection in Patients with Nonspecific Chronic Spinal Pain and Comorbid Insomnia: A Cross-Sectional Analysis
Source: J Clin Med. 2024 Mar 2;13(5):1452. doi: 10.3390/jcm13051452 (PMC10932262; doi:10.3390/jcm13051452)

**Figure S2**

*Centrality stability graph: GGM*

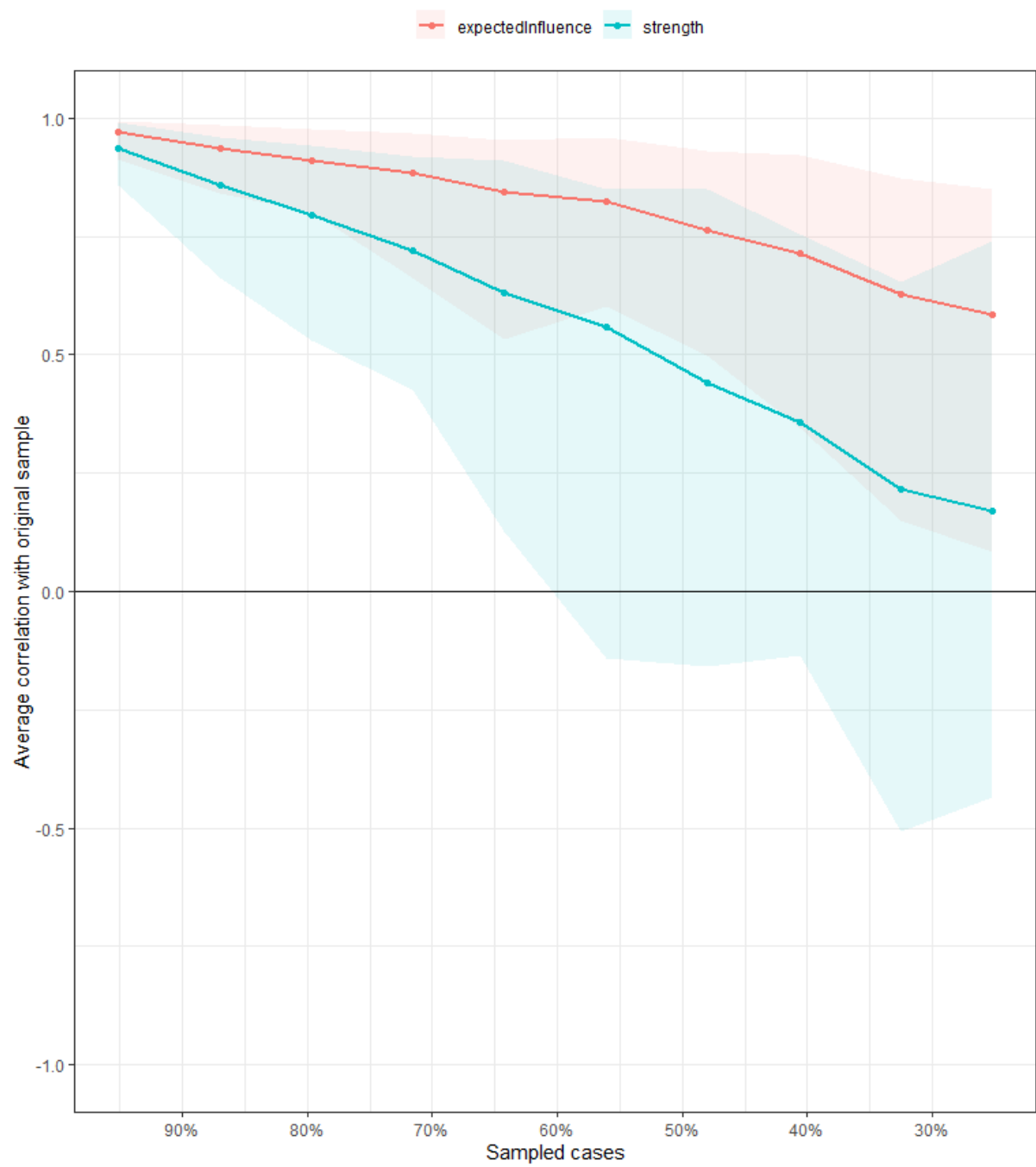

Supplement: Supplementary file 1 [file jcm-13-01452-s001.zip › Supplementary Figure S2.pdf]

**Figure S4**

*Spearman correlation network*

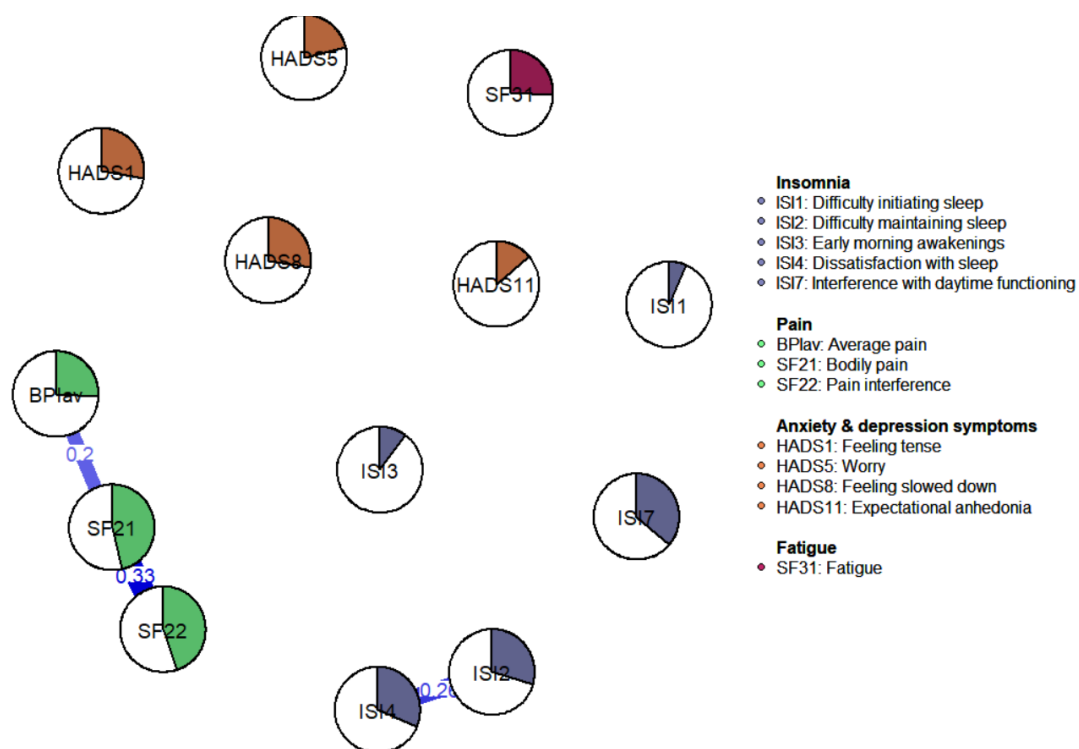

Supplement: Supplementary file 1 [file jcm-13-01452-s001.zip › Supplementary Figure S4.pdf]

Centrality difference test: GGM

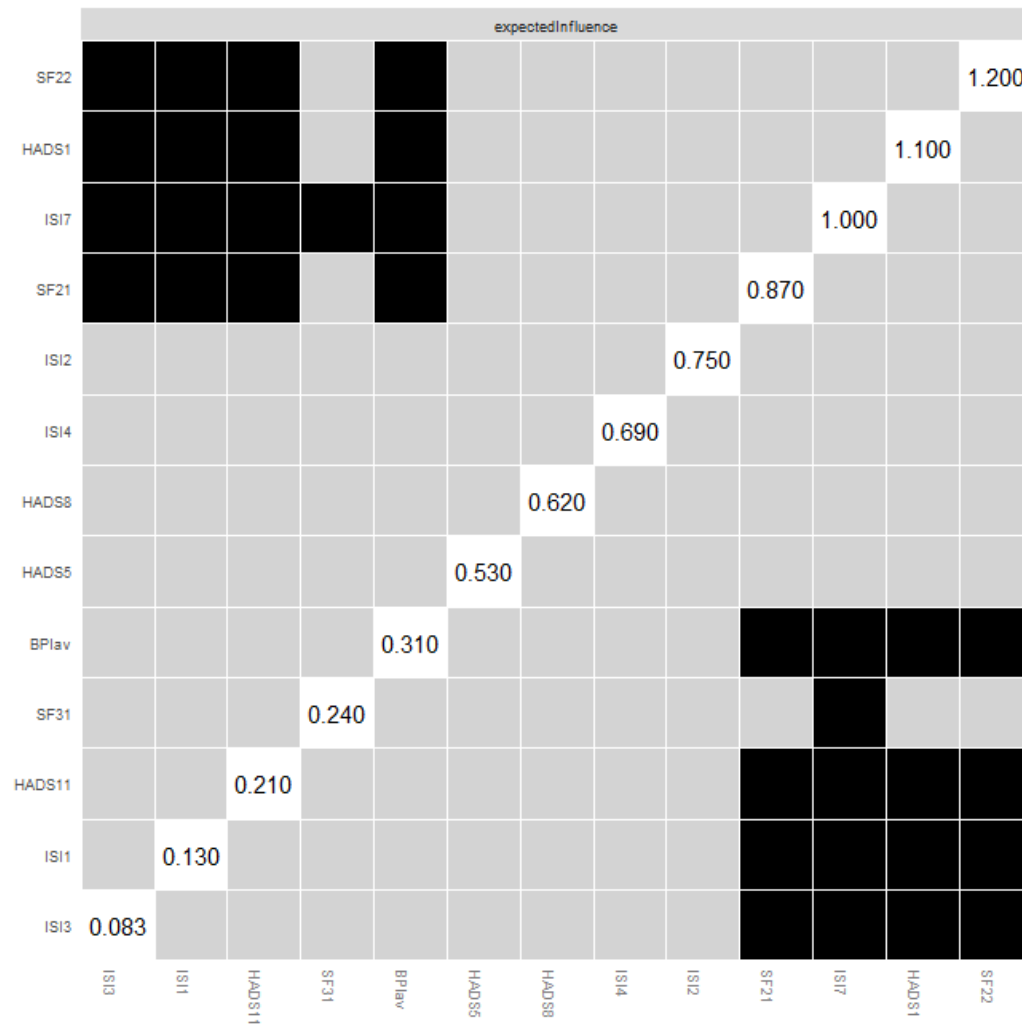

Supplement: Supplementary file 1 [file jcm-13-01452-s001.zip › Supplementary Figure S5.pdf]
